# Epilepsy Phenotype and EEG Finding of Rhythmic High‐Amplitude Delta With Superimposed Spikes (RHADS) in Succinate Dehydrogenase Deficiency

**DOI:** 10.1002/jmd2.70072

**Published:** 2026-02-01

**Authors:** Aaron B. Bowen, Chiadika Nwanze, Cesar Alves, Lance Rodan, Anna Lecticia Pinto, Melissa A. Walker, Irina Anselm, Phillip L. Pearl

**Affiliations:** ^1^ Department of Neurology Boston Children's Hospital Boston Massachusetts USA; ^2^ Division of Neuroradiology, Department of Radiology Boston Children's Hospital Boston Massachusetts USA; ^3^ Department of Neurology, Child Neurology Division Massachusetts General Hospital Boston Massachusetts USA

**Keywords:** complex II deficiency, epilepsy, Leigh syndrome, RHADS, succinate dehydrogenase deficiency

## Abstract

Succinate dehydrogenase (SDH) serves a dual function as complex II of the electron transport chain and an enzyme of the tricarboxylic acid cycle. Pathogenic variants in subunits of SDH result in diverse clinical presentations, including typically autosomal recessive neurodegenerative disorders. Biallelic variants in the SDHA subunit most often cause Leigh syndrome. However, epilepsy phenotypes of these patients are ill‐defined and there is only one prior report of epilepsy in a patient with SDHA deficiency. Here we report the seizure and EEG phenotypes of three autosomal recessive SDHA patients with refractory epilepsy, two of whom are siblings. These patients exhibit multiple seizure types and a variety of EEG findings, including a patient with rhythmic high‐amplitude delta with superimposed spikes (RHADS), a finding closely associated with polymerase gamma (POLG)‐related disorders.

## Key Points

- Patients with complex II deficiency may exhibit refractory epilepsy with multiple seizure types which may be accompanied by cortical abnormalities on MRI.
- Complex II deficiency may be associated with rhythmic high‐amplitude delta with superimposed spikes (RHADS), an EEG pattern typically associated with POLG disorders.

## 1 Introduction

Mitochondrial complex II, also known as succinate dehydrogenase (SDH), is unique in that it also serves a dual role as an enzyme in the tricarboxylic acid cycle. SDH is composed of four nuclear‐encoded structural subunits, including SDHA, a flavoprotein, SDHB, an iron sulfur‐containing subunit, and two membrane‐associated subunits, SDHC and SDHD. Pathogenic variants in SDH have a wide variety of clinical manifestations [1]. Heterozygous pathogenic variants in all subunits have been associated with hereditary tumor syndromes, including gastrointestinal tumors, pheochromocytomas, and paragangliomas (OMIM #614165). In contrast, complex II deficiency due to biallelic variants in subunits A, B, and D has been associated with multiple phenotypes, including Leigh syndrome [2, 3], leukodystrophy [4], encephalomyelopathy [5], and isolated cardiomyopathy [6]. A late‐onset neurodegenerative disease with optic atrophy, ataxia, and myopathy has also been reported with monoallelic pathogenic variants [7]. Variants in SDHA are more typically associated with Leigh syndrome. In contrast, variants in SDHB may share features with Leigh syndrome but often lack the characteristic basal ganglia lesions on neuroimaging [8]. Similarly, encephalomyopathy in patients with pathogenic variants in SDHD is often accompanied by normal neuroimaging studies [5]. Other common associated features include developmental delay/intellectual disability, cardiomyopathy, and ocular abnormalities (nystagmus, strabismus, optic atrophy).

In prior case series, SDH deficiency due to pathogenic variants in any of the mitochondrial disease‐associated subunits (SDHA/B/D) has been associated with the development of seizures in a minority of patients. Here, we report the epilepsy phenotypes of three patients with biallelic variants in SDHA, two of whom are siblings, representing the entire cohort of patients with SDHA deficiency currently followed at two tertiary pediatric academic centers. While the unrelated patient (patient #1) and the female sibling (patient #2) were both diagnosed with Leigh syndrome and cardiomyopathy, the male sibling (patient #3) manifested as refractory epilepsy and developmental regression without clear findings suggestive of Leigh syndrome on head CT. Furthermore, patient #1 was noted to have rhythmic high‐amplitude delta with superimposed spikes (RHADS), an EEG pattern often considered pathognomonic for POLG‐related disorders. This finding expands the phenotypic association of RHADS with mitochondrial disease to include Leigh syndrome due to complex II deficiency. Patient #2 exhibited a notched‐delta EEG pattern resembling a RHADS variant.

## 2 Case Presentation

### 2.1 Patient #1

A former full‐term male infant presented at 3 months of age with abnormal stiffening movements prompting administration of seizure rescue medicines. EEG and brain MRI at that time were unremarkable and no chronic anti‐seizure medicine was prescribed.

**FIGURE 1:**
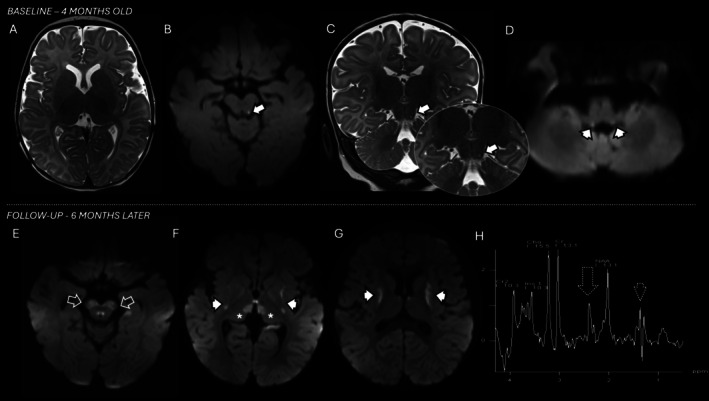
MRI progression for patient #1. Initial MRI brain performed at 4 months of age during presentation for cardiomyopathy and influenza infection (A–D) and follow‐up MRI and MRS (short echo‐time) studies 6 months later (E–H) after seizure onset. Selective lesions were noted at the onset involving the left inferior colliculus with reduced diffusivity (ADC MAP not shown) and T2 hyperintensity (arrows, B and C) and with reduced diffusivity along the vestibular nucleus in the inferior cerebellar peduncles (arrowheads, D). Progression with new lesions with reduced diffusivity (ADC MAP not shown) were noted in the follow‐up study including brainstem (open arrows, E), particularly in the cerebral peduncles and oculomotor nuclei, as well as in the mediodorsal aspect of the thalami (asterisks, F) as well as in the putamen bilaterally (arrowheads, F and G). Abnormal peaks of succinate and lactate are noted resonating at 2.36 and 1.3 ppm (dotted arrow and dotted arrowhead, H).

He presented 1 month later with lethargy and fever in the setting of influenza and was diagnosed with decompensated cardiogenic shock with severe left ventricular dysfunction and lactic acid of 6.2 millimole per liter. In urine, there were elevations of 3‐hydroxy‐3‐methylglutaric acid, 3‐methylglutaconic acid, succinic acid, fumarate, and malonate. Normal studies included plasma amino acids, acylcarnitine panel, and creatine kinase. EEG monitoring revealed intermittent right temporal slowing without epileptiform activity. Brain MRI was acquired showing small, focal lesions with reduced diffusivity in the left inferior colliculus and inferior cerebellar peduncles (Figure 1a–d). Rapid whole exome sequencing revealed biallelic variants of unknown significance in succinate dehydrogenase (paternally inherited c.1549 A>G p.K517E; maternally inherited c.373G>C p.D125H), and a vitamin cocktail with ubiquinone and riboflavin was started.

**FIGURE 2:**
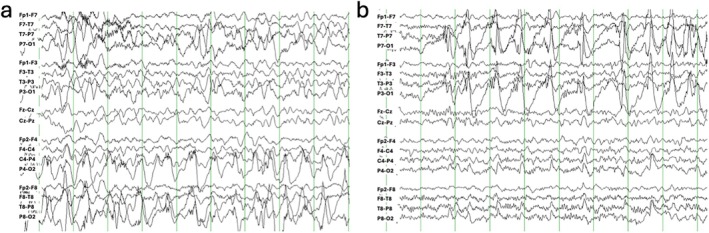
EEG showing RHADS in patient #1. (a) EEG recording obtained at 9 months of age around the time of seizure onset showing RHADS pattern over the right hemisphere before migrating to the left in panel (b) 44 min later.

At age 9 months, he developed episodic rhythmic shaking of the upper extremities and levetiracetam was started. EEG then showed frequent electrographic seizures originating from the right/left posterior quadrants, as well as RHADS (Figure 2). At 11 months, he developed episodes of desaturation and loss of tone with impaired awareness. EEG was similar with electroclinical seizures in the right mid‐posterior temporal region, intermittent right posterior quadrant slowing, and rare multifocal spike and wave discharges. Repeat brain MRI demonstrated new lesions with T2 hyperintensity and reduced diffusivity along the brainstem, thalami and striatum bilaterally (Figure 1e–g) consistent with Leigh syndrome. Short echo‐time MR spectroscopy showed a reduction in NAA to choline and creatine ratios, as well as abnormal lactate and succinate peaks (Figure 1h).

Around his first birthday, he developed status epilepticus due to a viral infection. Brain MRI revealed new areas of decreased diffusivity in the parasagittal frontal cortex and bilateral caudate nuclei. Several weeks later he developed focal motor status epilepticus with jerking of the left arm and face and numerous electrographic seizures. This evolved over several days to involve the contralateral (right) arm and face with inconsistent electrographic correlate, consistent with epilepsia pars continua (EPC). He was treated acutely with IV arginine during both episodes and then maintained on oral arginine. MRI showed normalization of cortical diffusion abnormalities in the frontal cortex along with resolution of seizure activity and EPC.

### 2.2 Patient #2 (Sibling)

This patient is a now 21 year‐old female born at 29 weeks gestation following pregnancy complicated by pre‐eclampsia and hemolysis, elevated liver enzymes and low platelets (HELLP). While in the NICU, she was diagnosed with cardiomyopathy and underwent muscle biopsy at 2 months of age which prompted a diagnosis of Leigh syndrome. However, the causative gene was not identified until 14 years of age when WES revealed biallelic variants in SDHA (maternally inherited c.91C>T (p.Arg31*); paternally inherited c.553C>G (p.Gln185Glu)).

Developmentally, she was able to slide on the floor and sit unassisted by age 3 years, although she never walked independently and does not communicate verbally. Her first known seizure occurred at 4 years of age with focal motor semiology (turn to left, head and face twitching), which has remained constant over the years. Seizures are daily and brief, lasting 5–10 s duration and have been refractory to multiple anti‐seizure medicines. She initiated the ketogenic diet at 12 years of age and received a vagus nerve stimulator at 14, but she continues to have daily seizures.

MRI at 9 years of age showed T2/FLAIR signal in the bilateral cerebellar hemispheres with vacuolization of the bilateral anterior medulla. EEG patterns have noted bifrontal slowing, 3 Hz generalized spike and wave discharges, multifocal spike and sharp discharges, myoclonic seizures (high amplitude spike with aftergoing slow wave), and at 11 years of age intermittent 3 Hz high voltage delta waves with sharp notches. MRI several months prior to this EEG showed bilateral cortical and subcortical FLAIR signal, thought to represent recent seizure activity.

### 2.3 Patient #3 (Sibling)

This patient, currently an 18 year‐old male, presented in the first year of life with missed developmental milestones, with independent gait at age 5 years and minimal expressive language restricted to signing. Seizure onset was at the age of 6 years and was associated with loss of ambulation. Semiologies included drop seizures with rapid loss of tone and head drops occurring up to 25 times per day. By age 14, seizures manifested as left‐sided hemiconvulsions, characterized by several daily episodes of left arm clonic–tonic activity and loss of lower extremity tone. Despite multiple anti‐seizure medicines, placement of a vagus nerve stimulator, and initiation of the ketogenic diet, he continues to have several seizures per day.

EEG revealed background generalized spikes and polyspikes. Atonic episodes were accompanied by 3–5 s bursts of rhythmic alpha frequency activity. While MRI was not performed in this patient to our knowledge, head CT was read as normal. A single focus of high attenuation in the left thalamus was noted and felt to be artifactual.

## 3 Discussion

We report a series of three patients with refractory epilepsy in the setting of biallelic variants in SDHA. Two of these patients were initially diagnosed with cardiomyopathy and subsequently Leigh syndrome. All three eventually developed refractory epilepsy with seizure semiologies including tonic–clonic, atonic, myoclonic, and focal motor including EPC. EEG showed various epileptiform patterns, as well as rhythmic high amplitude delta with superimposed spikes (RHADS) in one patient, and intermittent high voltage notched delta closely resembling RHADS in a second. RHADS is classically considered pathognomonic of hepatocerebral degeneration (Alpers disease) due to variants in *POLG* [9, 10]. RHADS has recently been described in a patient with myoclonic status epilepticus due to a pathogenic variant in the β2 subunit of the GABA‐A receptor, *GABRB2* [11], and in several cases of mitochondrial disease due to a variant in *DNM1L* [12, 13, 14]. In POLG, RHADS typically occurs in the setting of EPC with cortical involvement on MRI [15], which was also present in patient #1 with SDH deficiency, as well as the previously described cases with *GABRB2* and *DNM1L*. This suggests phenotypic overlap with POLG, which commonly involves cortical‐based “stroke‐like lesions” [16] and implicates cortical diffusion restriction on MRI in the pathophysiology of EEG RHADS. The mechanisms driving cortical pathology in POLG are incompletely understood, but these observations may reflect the susceptibility of cortical cells to certain types of genetic mitochondrial dysfunction.

Epilepsy is a relatively common feature of mitochondrial disease and Leigh syndrome specifically, affecting ~40%–50% of patients [17, 18, 19]. In Leigh syndrome, multiple seizure types have been described including generalized, focal, and myoclonic with variable inter‐ictal EEG findings such as focal or generalized slowing and epileptiform discharges. In contrast, limited information is available about the seizure types and EEG findings of patients with SDHA as many reported cases did not develop epilepsy. For example, a case report and review of seven prior cases, including 5 with Leigh syndrome, [2] showed only one case with epilepsy. This patient developed focal and generalized seizures and EEG at 9 months of age showed hypsarrhythmia [20]. Several patients with SDHB and D variants were found to have normal EEG, despite a history of seizure activity [5, 8]. The three patients reported here developed medically refractory epilepsy with a variety of seizure semiologies, suggesting epilepsy may be a prominent feature in this population, despite its absence in prior cohorts. Including the patients in this report, 3/7 (~43%) of patients with Leigh syndrome due to SDHA deficiency developed epilepsy, approximating the prevalence in the overall Leigh syndrome population. Although most described patients with epilepsy were also diagnosed with Leigh syndrome, patient #3 reported here did not have clear Leigh imaging findings. Thus, whether there are specific genetic, clinical or neuroimaging findings that predict the presence of epilepsy in complex II deficiency remains unclear.

Both genotypes reported here are novel. The variants in patient #1 were confirmed to be pathogenic based on Leigh phenotype and supported by the accumulation of succinate in urine as well as the brain via MR spectroscopy. In the siblings, the SDHA c.91C>T (p.Arg31*) variant is known to be pathogenic. Although SDHA c.553C>G (p.Gln185Glu) is classified as a VUS, there are no homozygotes in Gnomad and a very low allele frequency of heterozygotes. Furthermore, it has been associated with complex II deficiency phenotypes in ClinVar, including cardiomyopathy (RCV003473597.1) and pheochromocytoma/paraganglioma syndrome (RCV001070085.9). Thus, all the patients described here have convincing evidence for the pathogenicity of their associated variants and clinical phenotypes concordant with SDHA deficiency. To our knowledge, this is the first report of the EEG RHADS pattern in succinate dehydrogenase (complex II) deficiency.

## Funding

The authors have nothing to report.

## Consent

All patients have signed informed consent and agreed to be included in this report.

## Conflicts of Interest

The authors declare no conflicts of interest.

## Data Availability

Data sharing not applicable to this article as no datasets were generated or analysed during the current study.
